# Minimalist analogue robot discovers animal-like walking gaits

**DOI:** 10.1088/1748-3190/ab654e

**Published:** 2020-02-07

**Authors:** Benjamin J H Smith, James R Usherwood

**Affiliations:** bbab654e2 Royal Veterinary College, London, United Kingdom; 1 Author to whom any correspondence should be addressed.; bsmith@rvc.ac.uk; jusherwood@rvc.ac.uk

**Keywords:** robot, gait, biomechanics, locomotion, bioinspiration

## Abstract

Robots based on simplified or abstracted biomechanical concepts can be a useful tool for investigating how and why animals move the way they do. In this paper we present an extremely simple quadruped robot, which is able to walk with no form of software or controller. Instead, individual leg movements are triggered directly by switches on each leg which detect leg loading and unloading. As the robot progresses, pitching and rolling movements of its body result in a gait emerging with a consistent leg movement order, despite variations in stride and stance time. This gait has similarities to the gaits used by walking primates and grazing livestock, and is close to the gait which was recently theorised to derive from animal body geometry. As well as presenting the design and construction of the robot, we present experimental measurements of the robot’s gait kinematics and ground reaction forces determined using high speed video and a pressure mat, and compare these to gait parameters of animals taken from literature. Our results support the theory that body geometry is a key determinant of animal gait at low speeds, and also demonstrate that steady state locomotion can be achieved with little to no active control.

## 1. Introduction

The gaits that quadrupedal animals use have been a source of fascination for researchers for centuries, both from the perspective of biology and legged robotics. Understanding why animals use the gaits they do can reveal what parameters are selected for in legged locomotion, and may therefore lead to better treatments for gait disorders due to age or pathologies, and enable more robust and efficient walking and running robots.

A gait cycle is divided into the stance phase, where a foot is in contact with the ground, and the swing phase, where the foot is lifted and moved to the next position. Following Hildebrand [1], gaits can be categorised into symmetric, where the left and right feet of a pair move alternately (e.g. walking or trotting), and asymmetric, where the left and right feet move at approximately the same time (e.g. galloping or bounding). Symmetric gaits can be described using two quantities: duty factor; the proportion of the stride for which each foot is on the ground, and phase; the timing of the beginning of stance of a forefoot relative to the beginning of stance of the hind foot on the same side. For example, in a running trot the duty factor is  <0.5 (since the animal is completely off the ground for a proportion of the stride), and the stance and swing phases of diagonal pairs of legs are synchronised so the phase is 50%. It is generally agreed that gaits are linked to speed; typically, quadrupeds walk at low speeds, and transition to a trot and finally a gallop as their speed increases [2]. However, the mechanisms which determine which gaits are used in a particular situation, and how and why animals transition between gaits, are still poorly understood. Some animals, such as small rodents which use a running walk throughout most of their speed range [3], do not appear to have a strong relationship between their speed and their gait. Some species use rare or alternative gaits, such as camelids which pace at speeds where most animals might trot [4], or primates which use diagonal sequence walks where most animals would use a lateral sequence walk [5]. Conversely, some animals appear to avoid particular types of gait, such as gnu which transition directly from walking to cantering [6]. Some animals even use different gaits at different points in their lives, such as macaques, which have been observed to use lateral sequence walks when young, and shift to diagonal sequence as they age [7].

Both physical and neurological mechanisms have been proposed for why gaits are selected and how animals transition between gaits [8, 9]. One of the most popular theories is that quadrupedal animals select the gait which is energetically optimal at a given speed [10]; energy cost for a gait changes in a curvilinear way with speed, thus animals will switch gaits at the speeds where the energy curves for different gaits intersect, moving to whichever gait will minimise energetic cost. Recently, an energetic explanation has also been proposed for the phases used by walking quadrupeds[11]; the duty factor used by the animal determines the relative directions of the centre of mass velocity and the limb forces, and hence whether it is more economical to reduce vertical centre of mass velocity by distributing footfalls evenly through time (i.e. a phase of 25% or 75%) or to increase vertical centre of mass velocity with footfalls that occur close together (i.e. a phase closer to 50%). This is in contrast to previous stability based explanations for leg phasing during walking, which suggested that quadrupeds attempt to optimise their support polygon by avoiding situations where they would only be supported by two limbs [12]. As with bipeds, inverted pendulum dynamics impose an upper limit on the speed quadrupeds can walk before switching to a running gait [13]. Unlike bipeds, however, many quadrupeds exhibit a second gait transition from trotting to cantering or galloping; it has been suggested that this is because animals select the gait which minimises peak forces on the musculoskeletal system, and thus reduce the chance of injury [14]; peak forces tend to increase with speed in running, however there is a discontinuous drop in peak force when a quadruped switches from a trot to a gallop [15]. Recently, an alternative theory has been proposed for the trot-canter transition [16]; animals select gaits to avoid energetically disadvantageous pitching motions about their centre of mass.

As well as these mechanical explanations, a number of researchers [17–19] have also proposed that gaits can be modelled in terms of oscillator dynamics, with limb phasing and transitions between gaits emerging from the neural or software interactions of the oscillators. The biological basis for this model comes from work by Brown [20], who found that hindlimb rhythmic motor activity does not require sensory input. Later work by Grillner [21] found that sensory input can tune Central Pattern Generators (CPGs), but does not necessarily drive them, while Duysens and Pearson [22] found that applying a load to a leg can prolong the stance phase for an indeterminate length of time. One example of how this model can be implemented is given by Fukuoka *et al* [23], using a computer model of a quadruped robot with a CPG controlling each leg. These CPGs were connected by fixed couplings which had been tuned to achieve a steady trot; however, the CPGs could be adapted with feedback from force sensors on the model’s feet, which inhibited the leg from transitioning from stance to swing phase while the leg was loaded. This model was able to exhibit a range of gaits at different speeds, including walking, trotting and galloping, and transitions between the gaits. The researchers concluded that the pitch and roll of the body caused a difference in load between the legs of a diagonal pair; specific gaits are caused by a particular combination of body posture and speed. A similar principle was used by Maufroy *et al* [24] to control both locomotion and posture in both a modelled and real quadruped robot; each leg had its own independent CPG based controller, which set the leg into the stance phase when it was loaded, and into the swing phase when it was unloaded. Using identical parameters for all the legs resulted in a pace gait, where ipsilateral front and rear legs moved at the same time; incorporating a delay into the fore leg controllers resulted in a diagonal sequence walk, where a contralateral forefoot moved after a hind foot. Although the robot was originally designed with no interleg co-ordination, it was found to be sensitive to lateral perturbations, so an ascending co-ordination mechanism was implemented. The combination of CPGs and sensor inputs has also been used to improve robustness, with sensor inputs acting as reflexes which tuned the CPG to adapt to different situations; such as [25], where reflexes were used to tune a CPG to prevent stumbling over obstacles, and rolling on slopes.

Owaki *et al* [26] showed that the couplings between oscillators do not have to be encoded in the controller, and can instead be physical connections due to the body of an animal or robot, which they described as ‘physical communication’. They constructed a quadruped robot controlled using a central pattern generator (CPG) formed by four decoupled oscillators, one on each leg. A force sensor on each leg provided feedback to the oscillator, which was used to determine its phase. This robot, and its subsequent developments, was able to move with animal-like gaits, and carry out speed dependent gait transitions, despite the lack of centralised control [27]. As well as demonstrating that physical communication could be used to produce a quadruped gait in the absence of neurological coupling, these robots were also able to adapt to different loading configurations which changed the position of the centre of mass, by using different gaits, such as switching from lateral sequence to diagonal sequence walking. This suggests that body geometry plays a role in which gait is used. More recent research has found that the interdependence of body geometry and gait also extends to body bending; optimal gaits were discovered in a salamander-like robot when body flexion and leg occur in synchronisation [28]. The roles of other physical properties of a robot’s structure in determining gait have also been investigated, in particular compliance. The goal in [29] was to achieve fast stable locomotion via open loop compliant stabilization; this was achieved by adapting limb stiffness to speed in real time to follow the spring loaded inverted pendulum (SLIP) model, where minimum leg length was at midstance. This allowed the robot ‘Cheetah-cub’ to run stably at 6.9 body lengths per seconds, and traverse discontinuous terrain without needing any direct adaptation of its CPG controller. A similar concept known as ‘embodied computing’ was used in [30] to simplify the control of a quadruped robot; control signals were directly related to sensor inputs, via non-linear transforms which the robot learned in real time, and control was outsourced to compliant structural elements rather than a processor. This enabled the robot to discover stable trotting and walking gaits within a few strides.

The system presented here takes the principle of body structure affecting gait and extends it to a system in which gait is determined purely by body geometry, with no controlling oscillators or CPG. This is based on a recent paper which proposed that animals such as horses and sheep use a specific ‘grazing gait’ while foraging, with a footfall timing which emerges naturally from their body geometry [31]. The locomotion used during grazing differs from normal walking in that it has a duty factor approaching 1, rather than around 0.65, and a phase close to (but not exactly) 50%, rather than 25%. The intermittent nature of grazing requires the animal to move between postures that are statically stable (i.e. with at least three feet on the ground so that the centre of mass falls within the polygon of support), whereas the more dynamic nature of continuous gaits allows the animal to support itself on only two or one legs. Very slow walking, with a duty factor  >0.75, can achieve this stability requirement by moving a forefoot immediately after the ipsilateral hindfoot; however, this results in discontinuities in weight support, which may require corrections which are disadvantageous in terms of energy or stability. Instead, grazing animals move a foot when it is maximally unloaded; a gait which represents a local, rather than global minimum in terms of work, but which also minimises any disruption in weight support.

The grazing gait may be of interest to engineers interested in designing robots for moving intermittently over large areas, for example when surveying for minerals, or minesweeping; if low level locomotion control can be achieved with little to no computational effort then more processing power will be available for sensing and path planning. In a more general sense it adds further information to the discussion about the extents to which animal gaits are determined by neurological or mechanical structures.

## 2. Methods

### 2.1. Robot design

**Figure 1.**
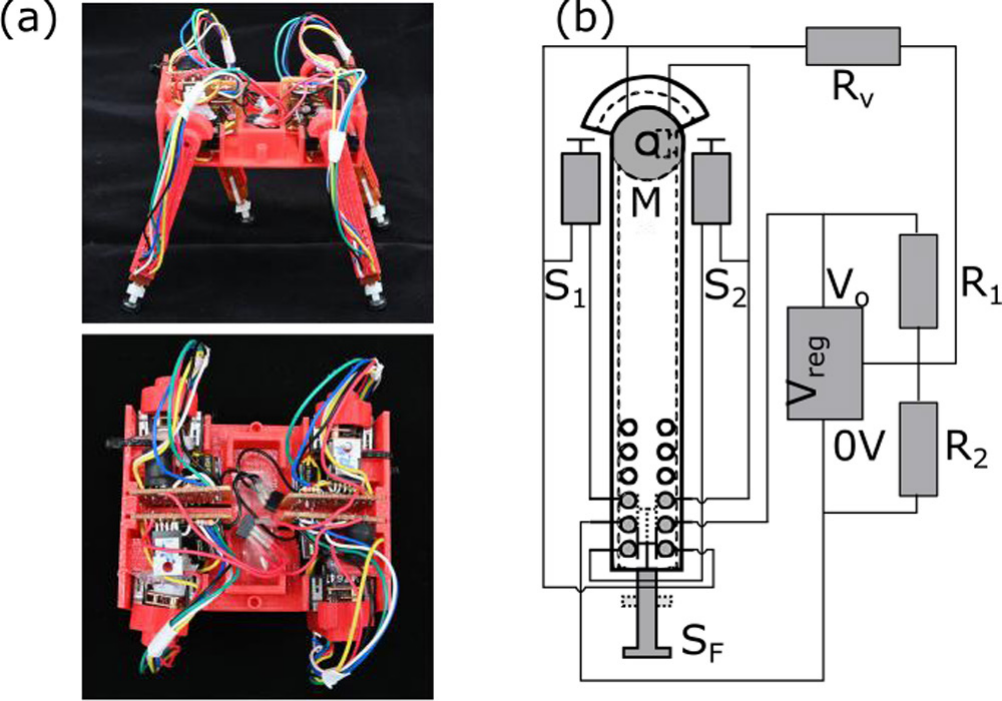
Robot design. (a) A photograph of the completed robot. Each of the legs is fitted with a hard rubber foot to aid traction. (b) Simplified leg circuit. The switch *S*_F_ determines the direction the motor M rotates, the switches *S*_1_ and *S*_2_ are limit switches, and the speed of rotation is determined by the output of the voltage regulator *V*_reg_, which is a function of the values of *R*, *R*_1_ and *R*_2_.

In order to replicate the model in [31] as closely as possible, the robot structure should meet the following assumptions: firstly, the hip and shoulder should vault from low to high then back to low again over the course of a stance; this requires constant leg length during the stance phase (i.e. no knee or ankle joints). Secondly, the shoulders and hips should be connected with a rigid, table top like linkage, which resists bending and twisting. The robot therefore consists of a rectangular body and four legs, each with one degree of freedom. Figure 1(a) shows a photo of the robot; it is 93 mm long, 92 mm wide (including the legs), stands 102 mm tall and weighs 220.1 g. More detailed mechanical and electronic plans can be found in the supplementary information S1.1 and S1.2 (stacks.iop.org/BB/15/026004/mmedia).

In order to demonstrate that biologically plausible locomotion can emerge primarily from the mechanical configuration of an animal with little to no neurological input, it was decided to build an analogue robot, controlled using analogue electronics rather than a digital microprocessor. Analogue robots include BEAM (biology, electronics, aesthetics and mechanics), or Biomorphic robots [32], which attempt to achieve biological-like reactions to stimuli using simple analogue circuits, and Braitenberg vehicles [33] which produce apparently complex behaviours such as exploring based purely on sensor inputs. These types of systems were particularly popular in the early days of robotics, when the size of computers made more complex on board controllers unfeasible; however, the close links between sensors and actuators with minimal interposition of software also makes them an interesting model of low level biological control. Still and Tilden demonstrated an analogue quadruped robot which was able to walk with no software [34]. Instead, control was provided using a ring of four coupled oscillators, each implemented using an inverter and a resistor–capacitor (RC) high pass filter. This network produced two different modes of oscillation, which translated to two different gaits: a walk like gait and a trot like gait; however, since the robot was only actuated by two motors its ability to mimic biological gaits was limited. Shaikh *et al* [35] used a Braitenberg vehicle to replicate lizard phonotaxis in a two wheeled robot; two microphones, filtered using their ear model, were coupled directly to the contralateral motor inputs. The output level from the ‘ear’ directly determined the speed of the motor, so that the robot would rotate to face, and then move towards, a stimulus of the correct frequency. This enabled continuous control of the robot’s motion, an improvement over the step control which resulted from having to make decisions at discrete intervals.

In the robot presented here, the analogue approach was carried out by using mechanical components such as switches, rather than encoders, and only analogue, rather than digital, components, so that the system moved purely on reflexes rather than logic, and the total number of electronic components was kept as low as possible. Each leg is controlled using an identical circuit (shown in 1(b)): actuation is provided by a 3 V brushed DC motor with an integrated gearbox with a high gear ratio (RS Pro 951D series) resulting in low speed, low power movements most closely analogous to the grazing gait, and low backdrivability and thus low compliance to better approximate the model. The DPDT switch S_F_ (Knitter MPBS-42H01-F14) is used to change the direction of leg movement; when it is depressed (i.e. when the leg is in stance), the leg moves backwards, and when it is released (i.e. when the leg is in swing) the leg moves forwards. The microswitches *S*_1_ and *S*_2_ (Omron D2F-FL3) on either side of the motor limit the distance the leg can move in either direction. Unloaded legs therefore move forward until either they reach the front limit, when they remain at the extreme forward position, or until they are loaded, when they move backwards until they either reach the rear limit, where they remain at the extreme backward position, or until they are unloaded. Power to all four legs is provided using a 9 V PP3 battery, due to its compact size. A step-down voltage regulator (ON Semiconductor LM2575TV-ADJG) is used to provide consistent voltage and current to the motor; the output voltage ${{V}_{o}}={{V}_{{\rm ref}}}\left(1+\frac{{{R}_{1}}}{{{R}_{2}}} \right)$, where *V*_ref_ is the battery output and *R*_1_ and *R*_2_ are as shown in figure 1(b). By connecting the resistor *R*_*v*_ across one side of the *S*_F_ switch, it changes between being connected in parallel across *R*_1_ or *R*_2_ when *S*_F_ toggles. This makes it possible to change the effective value of the ratio $\frac{{{R}_{1}}}{{{R}_{2}}}$ for each different state of *S*_F_, and thus achieve different output voltages from the regulator depending on whether the switch is depressed or not. This results in faster movements when the leg is unloaded, minimising the swing time, while allowing for slower, higher torque movements in stance. However, as there is no feedback control, the instantaneous speed of the motors is dependent on motor mechanical load.

The body and legs were designed in Siemens SolidEdge and 3D printed in PLA using a MakerBot Replicator 2. Each leg shares a similar basic design; however, the ‘shoulders’ which contact the limit switches are asymmetric such that the forward reach of the front limb is mirrored in the backwards reach of the ipsilateral rear limb, and the backwards reach of the front limb is mirrored in the forwards reach of the ipsilateral rear limb. This prevents the feet colliding during walking. A large portion of each leg is made up of *S*_F_ which acts as a contact sensor; these switches were modified from an off the shelf model to have a softer and shorter spring that would be deflected by the relatively low mass of the robot, and to reduce the travel of the switch so as to minimise leg compliance which would diverge from the idealised ‘toppling table’ model. The length of the legs was also designed to prevent collisions, and also to ensure that the ‘toppling’ motion caused large enough vertical displacements to unload or load the switches at the start or end of stance.

### 2.2. Experimental protocol and data analysis

A VH3 Walkway pressure mat (Tekscan Inc., Boston, MA, USA) was used to determine the forces exerted by all four of the robot’s feet concurrently, while a Basler acA2000-165umNIR high speed camera (Basler AG, Ahrensburg, Germany) was used to video each trial. A push switch connected to a USB-6008 DAQ (National Instruments, Austin, TX, USA), which sent a pulse to both instruments, was used to trigger simultaneous data collection via Tekscan Walkway software v7.70 in the case of the pressure mat, and custom LabView code in the case of the camera; both sets of data were collected at 100 Hz. The robot was placed at one end of the walkway, slightly before the edge of the sensing area. Recording was triggered when the robot reached the recording area, and stopped when the robot reached the end of the mat or stopped moving (e.g. because it toppled over). The best 25 videos were used for analysis. Initially the robot was too light to produce usable data from the pressure mat, so 50 g of wheel balance weights were fixed to the underside of the body, as close to the centre as possible.

## 3. Results and discussion

Tekscan Walkway software v7.70 was used to segment the pressure mat output into individual footfalls and calculate vertical ground reaction forces (GRF), Kinovea 0.8.15 was used to digitise the high speed videos and MATLAB was used to analyse the digitised data. Stride time for each leg was defined as the time between two consecutive touchdowns. Duty factor was calculated for each leg as the duration of contact divided by the stride time. Phase was calculated for left and right sides separately as the interval between hind and fore foot touchdown times divided by median stride time for that side. Since strides did not always start with the same foot, phases were converted to radians and transformed using the MATLAB function ‘wrapTo2Pi’, for analysis; normalised values are reported here for ease of comprehension.

**Figure 2.**
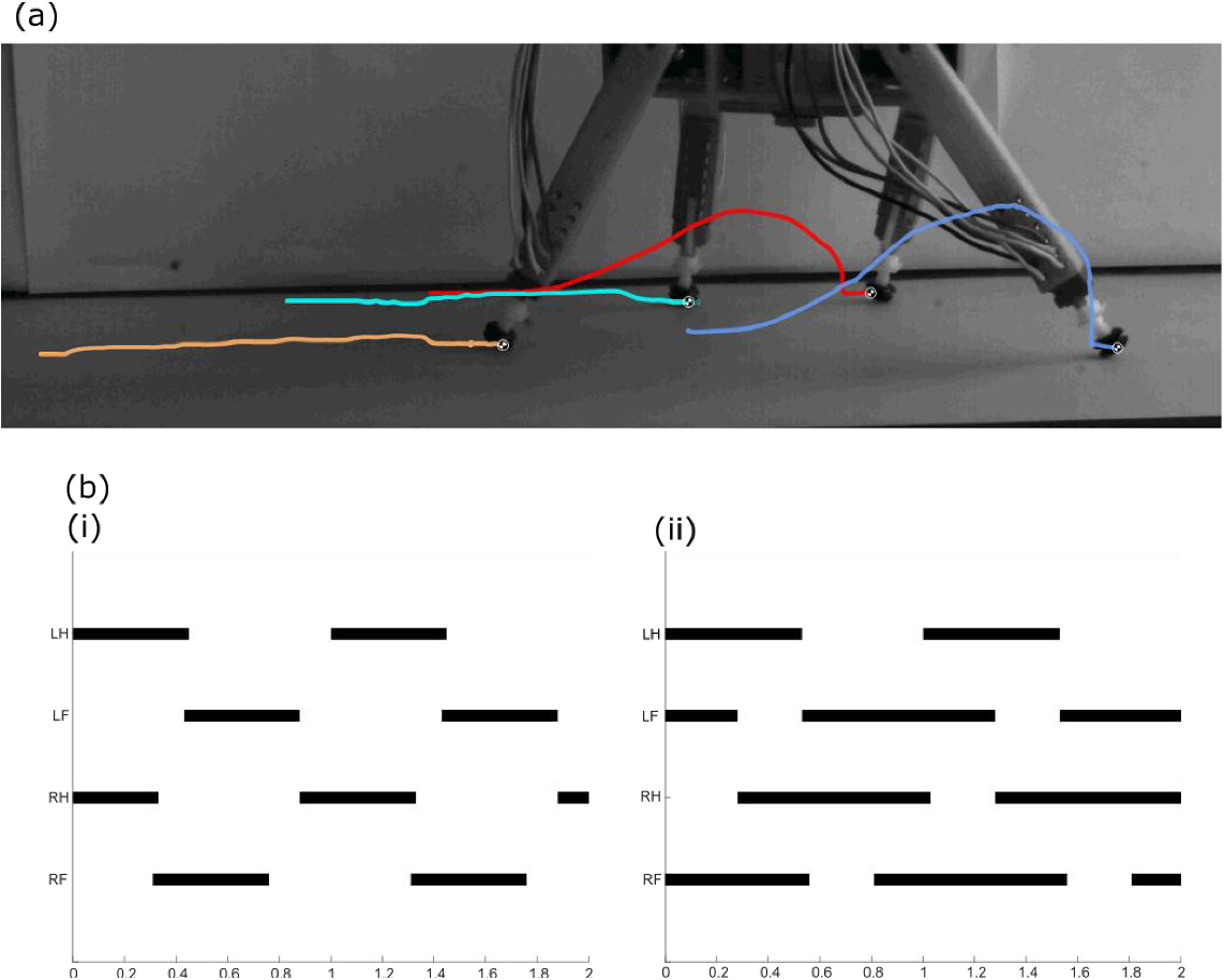
Exemplar kinematics for the robot’s gait. (a) High speed video frame showing trajectories of the robot’s feet throughout a stride, tracked using Kinovea. (b) Gait diagrams showing how the robot’s walk changes with duty factor. (i) a duty factor of 0.45 m and a phase of 0.43, (ii) a duty factor of 0.75 and a phase of 0.53.

Figure 2(a) shows an example of the trajectories taken by the robot’s feet; hind and forelegs display very different trajectories. The foreleg trajectories are asymmetric, initially with little vertical displacement, then lifting higher towards the end of the swing. Conversely, the hind leg trajectories are more symmetric, but with very little vertical displacement. Further examples of the robot’s motion can be found in videos S2.1–2.3 in the supplementary information, along with corresponding pressure mat output. The order in which the feet move is very consistent; 82.8% of the foot transitions are from either hind foot to ipsilateral forefoot, or forefoot to contralateral hind foot, the same as in the lateral sequence walk used by most quadrupedal animals. In contrast, the stance and stride times, and hence the duty factors, display a lot of variation; average means and standard deviations are shown in table 1 below.

**Table 1.** Mean and standard deviations of stance time, stride time and duty factor for each of the legs.

	LH	LF	RH	RF
Stance time (s)	0.82 ± 0.18	0.82 ± 0.36	0.88 ± 0.23	0.65 ± 0.25
Stride time (s)	1.51 ± 0.24	1.23 ± 0.33	1.51 ± 0.20	1.31 ± 0.42
Duty factor	0.54 ± 0.10	0.64 ± 0.15	0.60 ± 0.11	0.49 ± 0.12

**Figure 3.**
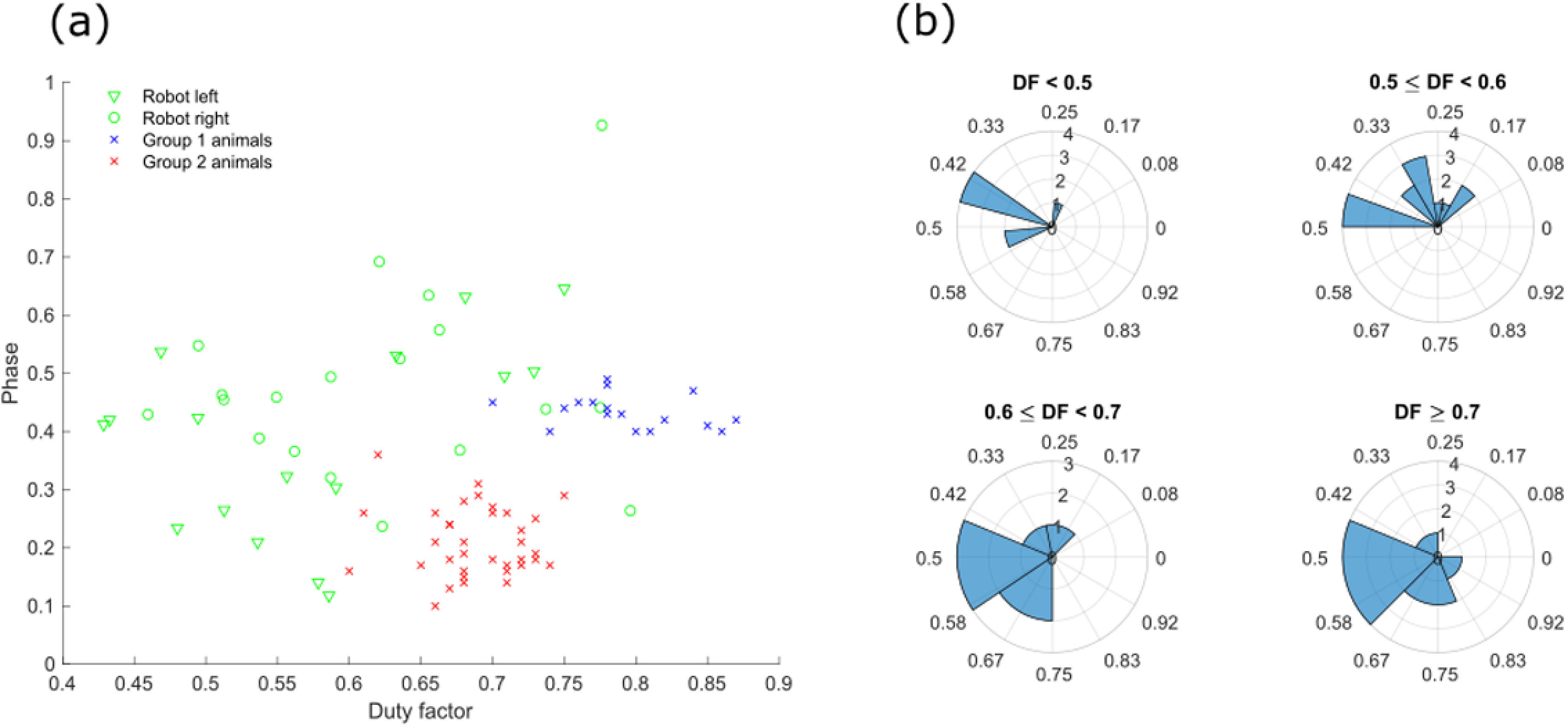
The relationship between phase and duty factor (calculated as described above) in walking animals and the robot. (a) Phase for left and right sides plotted against median duty factor for that side, for individual strides. Values for animals and group labels are taken from [11]; group 1 animals are slow or small and use high duty factors (e.g. hippo, mouse), while group 2 animals are more upright and use lower duty factors (e.g. horse, dog). (b) Rose plots of phases binned by duty factor, DF, illustrating how phases rotate around counterclockwise as duty factor increases.

This variation in foot timings is likely a consequence of the lack of active control making the robot very sensitive to initial conditions. The robot is lifted so that all four feet are unloaded and return to their extreme forward position, then it is placed on the mat with all four feet making contact almost simultaneously; despite this, slight variations in foot loading or relative leg position occur at the start of a trial, which then propagate through the walk cycles. Since there is no feedback control in the motors, neither the speed of leg movements, nor the speed of the whole robot is constant throughout, or between strides. The fact that the same foot order is used in almost every transition, despite these variations, suggests that it is not dependent on stance kinematics or overall body speed, and is conversant with the model presented in [11, 31], where the relationship between phase and duty factor is independent of absolute speed. However, one effect of the changes in timings is that while the order of leg movements is the same, the relative phase of the leg movements changes with duty factor, as shown in figure 3(a). This means that at duty factors up to 0.6 the phase is mostly below 0.5, however as duty factor increases the phase also increases. This can be seen more clearly when the data is binned by duty factor and plotted as rose plots (figure 3(b)); as duty factor increases the distributions rotate counter clockwise from the upper quadrants to the lower left quadrant, with a modal phase of 0.43 at duty factors less than 0.5, to a modal phase of 0.53 at duty factors higher than 0.7. Gait diagrams for these two cases are illustrated in figure 2(b). The overall trajectory of the duty factor—phase plot follows that of the animals presented in [11], but shifted so that phases are higher for a given duty factor. Towards the higher end of duty factor and phase it begins to extend into the region occupied by primates (which typically use a diagonal sequence walk close to 0.75 phase); however, diagonal transitions (i.e. from hind to contralateral fore, or fore to ipsilateral hind) only comprised 7.4% of the total number of the robot’s foot movements. The gait used by the robot is more similar to the grazing gait defined in [31], to the extent that it has a conserved footfall pattern with variations in stance time, and phases close to 0.5, although these characteristics are also displayed at duty factors much lower than 1, and the phases do not appear to be converging on 0.5, instead overshooting, particularly at higher duty factors. Unlike the grazing gait, the robot’s gait is not truly intermittent; while some legs achieve high duty factors above 0.8 and others achieve low duty factors below 0.5, this is typically due to high levels of rolling to one side or the other, rather than pauses where all four legs are on the ground for high duty factors, or aerial phases for low duty factors. If the robot entered either of these states it would likely signal the end of the trial, either because it would remain stationary, or because it would tip over (depending on whether the positions of the feet enclosed the projection of the centre of mass to the floor when they were all loaded). This tendency to roll may also explain why the phases did not converge on 50%; the simplified model of the grazing gait considered only displacement of the body and feet in two dimensions—vertical and front-rear; however, the rolling moment also produces lateral movement of the centre of mass (c.f. [36]). At the moment of foot transition in a pure 50% phase gait, the feet on one side of the robot are at their point of closest separation, while the feet on the other side are at their furthest separation. This means that the robot is particularly vulnerable to excessive rolling, and potentially tipping over sideways, resulting in a failed stride which was therefore not included in the analysis.

**Figure 4.**
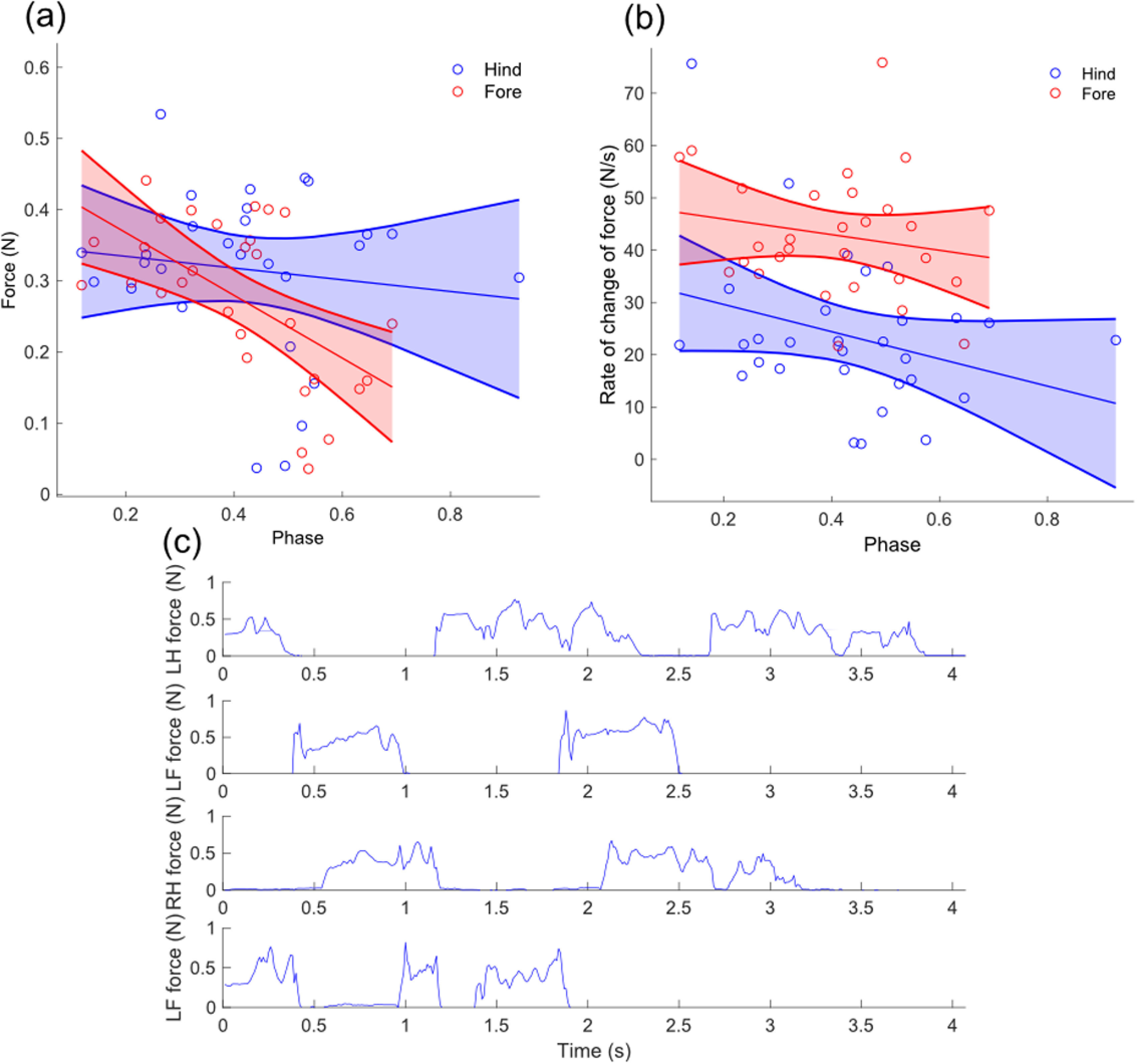
Forces exerted by the robot’s feet, thresholded to remove noise (a) Mean forces for hind and fore feet plotted against phase. (b) Peak (maximum) rate of change for hind and fore feet plotted against phase. (c) An example force trace for all four legs: LH denotes left hind foot, RH denotes right hind, LF denotes left fore and RF denotes right fore. Discontinuities can be observed at the point of touch down in the traces for the fore feet.

Figures 4(a) and (b) show mean force, and peak rate of change of force throughout stance plotted against phase for fore and hind legs. A threshold of 25 mN was applied to remove noise; this level was determined by recording data from the unloaded pressure mat, with the threshold set to one standard deviation above the mean noise value. Mean forces are similar for fore and hind feet across phases, with a decrease in peak force as phase increases, particularly in the fore limbs, which is likely due to the corresponding increase in duty factor [37]. However, rate of change of force is much higher for fore limbs; this corresponds to the trajectories shown in figure 2(a), where the vertical displacement in the forelimbs changes much more sharply than in the hind limbs. For both hind and fore limbs peak rates of change of force decrease with increasing phase. Figure 4(c) shows a typical force trace over a stride for each of the legs, and demonstrates that the high rate of change of force in the forelegs is due to a large, but transient, spike in force at the beginning of stance. This corresponds to the discontinuities identified in [31]; the reduction of these discontinuities at higher duty factors and phases supports the theory that the grazing gait is used to minimise disruptions in weight support, and although the order of foot movements is not the same as primates, it also suggests that using higher phases may be a way to reduce discontinuities which would be undesirable when walking on branches. The relative timings of fore and hindfoot movements also support leg loading over the stability theory; if the legs were moving in a way that maximised stability margin, then a hindfoot movement would be followed immediately with a forefoot movement, while the fore-hind transition would have a longer interval. This would result in the feet forming a parallelogram of support. However, the mean time interval between fore-hind foot movements 0.62  ±  0.33 s, much shorter than the mean interval of 1.01  ±  0.44 s between hind-fore movements, and much closer to the grazing gait where the hind limb moves directly after the forelimb, forming isosceles trapeziums of support.

## 4. Conclusions

In this paper we have demonstrated that it is possible to achieve quadrupedal walking using only reflexes responding directly to sensor inputs, without any form of directed software controller. Furthermore, we have found that this gait is similar in a number of ways to gaits found in nature; the lateral sequence walk used by most animals at lower duty factors, and the ‘grazing gait’ used by many ungulates while feeding at higher duty factors. These results provide some empirical support for the theory proposed in [31]: that the grazing gait develops spontaneously from an animal’s body geometry, with the timing and order of foot movements determined by leg loading rather than attempting to maximise stability. It was also suggested in [31] that the ‘toppling table’ model could also be applied to primate gaits which have evolved in response to the challenges on moving on an arboreal substrate; higher phases may be a tactic to ensure that any discontinuities in force occur at hind limb placement (when the limb contacts substrate that has already been tested) rather than forelimb placement (when the limb contacts new substrate). The results presented here may also support this hypothesis, by revealing that higher phases can reduce discontinuities in force. Future manipulations, such as changing the position of the robot’s centre of mass, or changing the ratio of leg to body length, could be used to test how well the ‘toppling table’ theory holds for more specific body morphologies, such as humped animals like camels.

The similarities with the model in [31] occur despite the fact that it was impossible to reproduce the model’s motion exactly. For example, there is no swing phase in the model, legs simply disappear at the end of stance and reappear at the beginning of the next stance; the feet of the model remain stationary during stance, whereas in the robot there is some slipping; the limbs in the model move at a constant speed, whereas in the robot the speed is dependent on limb loading; and the model reacts to changes in loading instantaneously, whereas the robot’s reactions are limited by the physical delays inherent in its mechanical and electronic components. Although these factors may be the cause of the variations observed in stance and swing times, they did not prevent the robot reproducing the gait used by the model, suggesting that geometry dominates over them in terms of determining the robot’s gait.

From a robotics perspective, our results contribute to the literature on walking robots with distributed, embodied or reflexive control schemes by showing that consistent walking gaits do not need to be encoded using CPGs or oscillators, and can instead emerge purely from body geometry and hardware dynamics. While some controller oversight would be necessary for speed and direction control and perturbation rejection, the computational load would be significantly reduced by removing the need for continual step to step control.
